# Commissioning of self-management support for people with long-term conditions: an exploration of commissioning aspirations and processes

**DOI:** 10.1136/bmjopen-2015-010853

**Published:** 2016-07-15

**Authors:** Claire Reidy, Anne Kennedy, Catherine Pope, Claire Ballinger, Ivo Vassilev, Anne Rogers

**Affiliations:** Faculty of Health Sciences, University of Southampton, NIHR Collaboration for Leadership in Applied Health Research (CLAHRC) Wessex, Southampton, Hampshire, UK

**Keywords:** Commissioning, Health services research, Self-management support, Ethnography

## Abstract

**Objectives:**

To explore how self-management support (SMS) is considered and conceptualised by Clinical Commissioning Groups (CCGs) and whether this is reflected in strategic planning and commissioning. SMS is an essential element of long-term condition (LTC) management and CCGs are responsible for commissioning services that are coordinated, integrated and link into patient's everyday lives. This focus provides a good test and exemplar for how commissioners communicate with their local population to find out what they need.

**Design:**

A multisite, quasi-ethnographic exploration of 9 CCGs.

**Setting:**

National Health Service (NHS) CCGs in southern England, representing varied socioeconomic status, practice sizes and rural and urban areas.

**Data collection/analysis:**

Content analysis of CCG forward plans for mention of SMS. Semistructured interviews with commissioners (n=10) explored understanding of SMS and analysed thematically. The practice of commissioning explored through the observations of Service User Researchers (n=5) attending Governing Body meetings (n=10, 30 hours).

**Results:**

Observations illuminate the relative absence of SMS and gateways to active engagement with patient and public voices. Content analysis of plans point to tensions between local aspirations and those identified by NHS England for empowering patients by enhancing SMS services (‘person-centred’, whole systems). Interview data highlight disparities in the process of translating the forward plans into practice. Commissioners reference SMS as a priority yet details of local initiatives are notably absent with austerity (cost-containment) and nationally measured biomedical outcomes taking precedence.

**Conclusions:**

Commissioners conceptualise locally sensitive SMS as a means to improve health and reduce service use, but structural and financial constraints result in prioritisation of nationally driven outcome measures and payments relating to biomedical targets. Ultimately, there is little evidence of local needs driving SMS in CCGs. CCGs need to focus more on early strategic planning of lay involvement to provide an avenue for genuine engagement, so that support can be provided for communities and individuals in a way people will engage with.

**Strengths and limitations of this study**

- As a study taking place 14 months on from the establishment of Clinical Commissioning Groups (CCGs), it provides a snapshot of how these organisations commission SMS at a time of flux and change.
- This quasi-ethnographic approach uses data from a number of sources: documentary analysis, interviews and observation, which enhances the strength of the findings, (although it is relevant to note that some data were missing from some sites).
- Exploring the public-facing messages and descriptions that CCGs portray about self-management and aligning this with the experience of CCG Governing Body meetings which occur ‘in public’ allow for a novel demonstration of how the message that is given to the public plays out in practice.
- The work was undertaken in one region and therefore may have limitations in terms of typicality and representation of the full range of variation in all English CCGs.

## Introduction

This study seeks to explore how self-management support (SMS) is being understood and made available to patients through local commissioning. In 2013, Clinical Commissioning Groups (CCGs) were created by the Health and Social Care Act 2012 (HSCA12) reforms that were intended to bring decision-making closer to the front line. SMS has been declared a priority as an essential element of integrated systems of support for long-term conditions (LTCs)^1–3^ and a means of achieving cost-containment. SMS that involves the actions and activities of patients themselves has been linked to a health service agenda of more inclusive patient and public involvement (PPI),^4^ an ethos which is also reflected in the new guidance of how CCGs should operate.^2^ ^5^ ^6^ Thus, the extent of engagement and participation of patients and the public in CCGs is a good indicator of the extent to which CCGs are progressing with a SMS agenda and makes it different from other areas of commissioning, because patient actions are a central element to the success of implementing local SMS strategies and interventions. The focus of the study reported here explores how SMS has been conceptualised by commissioners, how this commitment works through into practice (in terms of decisions made by CCG Governing Bodies and commissioners), and to what extent commissioning decisions are made through engagement with patients and the public (as a means to develop locally appropriate services).

SMS constitutes one of the top 10 priorities for transforming the healthcare system.^3^ SMS is one means through which health and social care services can enable people to take ‘better care’ of themselves^7^ and encourages the assumption of responsibility by individuals for making decisions to optimise health and well-being. SMS traditionally involves increasing the capacity, confidence and efficacy of the individual to self-manage by providing a range of options. Self-management (SM) for LTCs includes the actions and resources people use to meet physical, social, emotional and psychological needs, which affects: response to symptoms; effective working with health professionals and mobilisation of community resources. SMS has been viewed as necessary for; improving health outcomes, ensuring appropriate utilisation of services, increasing patient confidence, reducing anxiety, reducing unplanned admissions, improving medication and treatment adherence and reducing health systems cost.^8–11^ The SMS schemes, which have been developed and implemented in the UK over the past 20 years, view the patient as the expert in their condition (eg, The Expert Patients Programme)^12^ and the ethos of patient's voice and choice is evident in the development of recent provision, which has included: new technologies, patient information provision, skills training, support from health professionals and the promotion of the mobilisation of resources from personal support networks.^2^ ^5^ ^13–16^

### The commissioning process, integrated care and why SMS is a priority

To date, there has been little research attention paid specifically to the new commissioning arrangements for how the principles of SMS provision have been translated into practice by commissioning bodies, with previous research largely focusing on the organisation of commissioning arrangements and the attendant contracting and transactional processes.^17–22^ CCGs are scrutinised and monitored as commissioners of health provision in England, with the intention of extending their remit to jointly commission social care alongside local authorities under the governments' integrated care agenda. National Health Service (NHS) England, the national body who oversees the NHS budget, has celebrated the integrated care agenda as a ‘person-centred’, whole-system approach of collaborative working and aligning resources to help people self-manage more effectively at a time of fiscal restraint in the NHS.^5^ Integrated care has been considered by NHS England as integral to the change and adaption needed to meet the future challenges of a growing population living with LTCs with; patient led commissioning, increased choice and personalised care as central to this change.^2^ ^7^ ^10^ ^23–25^ One of the means by which NHS England has championed integrated care is through creating Vanguard sites; healthcare providers chosen to support improvement and integration of services, with the aim of providing inspiration to the rest of the health and care system. Such sites are supported financially and practically through NHS England.^14^ Integrated care for people with LTCs is intended as a focus of those responsible for commissioning services, and with it increasing attention has been placed on maximising the potential of SMS as a way to use NHS resources more efficiently while demand for healthcare is rising. The Wanless report into NHS resource requirements identified effective SM as an essential part of the ‘fully engaged’ scenario, which it predicted would bring about the greatest gains in public health for the least cost and this has been reinforced in subsequent policymaking with regard to LTC management.^4^ ^26^

**Figure 1:**
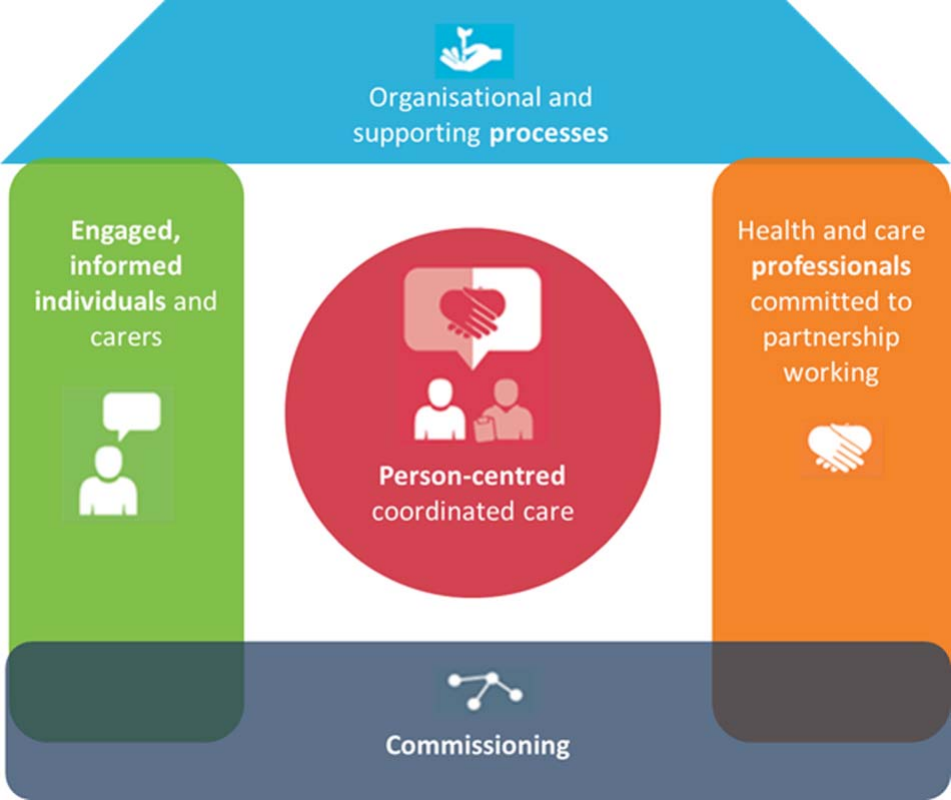
The House of Care Model (NHS England).

However, effective LTC management requires SMS that can be built into everyday life. This relies on considering the patient's social and cultural background as it is from this background that patients interpret and act on decisions about their treatment and recovery.^15^ Thus, CCGs are encouraged by NHS England to use the ‘House of Care’ model (figure 1),^2^ which represents a move away from the traditional ‘Medical’ model of health service provision and focuses instead on the integration of service users' experiences and resources. This has been seen as a way of re-distributing burden on the health services by managing the gap between the supply of health services and the demand from patients (‘demand management’).^27^ However, a crude focus on ‘demand management’ can sit in tension with involving patients and the public as partners in care; a lack of sensitivity to how patients use information, what information they need and the mechanisms and support they personally require to enable them to look after themselves could lead to ineffective SMS interventions being implemented. Imison and Gregory^28^ suggest that it would be unwise to solely focus on ‘demand management’ and rather, this should be seen as part of a wider strategy for maximising value from the NHS budget while focusing more on enabling patients to make informed decisions by maximising shared decision-making and utilising patient feedback measures. Effective SMS, therefore, requires listening to the patient voice, to avoid services being implemented that do not actually meet the needs of patients. Although, how much local commissioners are actually listening to the patient voice is unknown.

### Engaging the patient and public voice: commissioning personalised care

The ‘no decision about me without me’ commitment from the government^13^ is focused on shared decision-making, and pathways for patients and the public to influence commissioning decisions are a key part of the intended process. But, while PPI is seen as needing to be represented in policymaking and the operationalisation of SMS,^15^ it is unclear how this is perceived and acted on in the commissioning deliberations and decisions of CCGs. SMS relates directly to the need for services to be tailored to the patient and, thus, if decisions about such services are made without genuine collaboration with patients and the public, then services are likely to add to failed SMS services that have gone before them. In supporting people to participate in healthcare decisions, whether through partnerships with professionals or engaging with the commissioning process, CCGs need to provide access to information which can help their population make better decisions about their care.

One of the key goals of the reforms under the HSCA12 was to increase the public accountability of those responsible for commissioning care for patients (CCGs).^22^ ^24^ ^29^ NHS England published a guide for CCGs in December 2013, justifying planning for patients at a local level and requiring CCGs to develop a 2-year Operational plan and 5-year Strategic plan.^30^ However, our earlier work reviewing the plans of the (at that time) 211 CCGs in England indicated that 2 years down the line there were varying degrees of transparency in the work of CCGs,^31^ and that not all CCGs were providing their local populations with access to information that could help them make better decisions about their care. This work included regional disparities where some CCGs, largely in northern and more deprived parts of the country, provide less easy access to their forward plans in comparison to more affluent CCG localities with smaller populations.

Since SMS is so directly linked to the day-to-day lives of people, it is an example of commissioning decisions that most obviously require PPI input, so that support can be provided for communities and individuals in a way that people will engage with. Here, we explore commissioners' understanding and perception of local needs and SMS, as well as how they translated their understanding into actions and objectives that were commissionable alongside assuring local people that local services meet their needs. This study examines how this is played out in practice and the range of voices that are actually being involved in the development of NHS SMS services. As part of this study, we have used methodological innovation^32^ in working alongside and supporting patients and the public as Service User Researchers (SURs).

This study focuses on CCGs in the areas surrounding the National Institute for Health Research Collaboration for Leadership in Applied Health Research and Care (NIHR CLAHRC) Wessex, the south coast of England. The NIHR CLAHRC Wessex is a research and implementation programme which runs over 5 years, with the aim of improving the health of the people of Wessex and the quality and cost-effectiveness of healthcare.

## Methods

### Study design

We conducted a multisite, quasi-ethnographic analysis of nine CCGs in the south of England (table 1) to explore the ‘new’ NHS structure of commissioning relating to the implementation of SMS services. The study was undertaken over 12 months from June 2014 to May 2015. Data collected within each phase are detailed in table 2. An overview of the study is shown in table 3. Ethnography, and specifically direct observation, has been found to be particularly suited to uncovering the structural features of ‘new wave’ public policies, of which commissioning following the HSCA12 is one.^20^ Here, it allows for a comparison of the blueprint of the NHS with narrative accounts of SMS and patient engagement, and actual observations of decision-making and promotion of commissioner priorities to elicit how these priorities are enacted in routine public-facing meetings. The study comprised case studies of the nine CCGs and had three phases: the collection and analysis of documents (official 2-year Operational and 5-year Strategic plans of CCGs—both plans were sought from all nine CCGs); semistructured interviews with commissioners (commissioners from all nine CCGs were invited to participate) by one researcher (CR, EB, JE) and observations of CCG Governing Body meetings (which are held in public) by public and patient representatives (SURs) and researchers (CP, CA and CR). Phase I explored the aspirations and priorities of CCGs in commissioning SMS; phase II illuminated commissioners' conceptualisations of SMS initiatives, whereas phase III sought to elucidate how commissioning intentions for SMS play out in practice in a, supposedly, public setting.

**Table 1:** Demographics of CCGs in the south of England^iv^

CCG name	Population 2014* (Av† 266 525)	Practices (Av†=38)	Running cost allowance £m (1.66–21.75)	Revenue allocation 2013–2014 £000	IMD score‡,* (Av†=22.07)
CCG 1	270 070§	37¶	6.38¶	272 132	26.88§
CCG 2**	197 335¶	21¶	4.91¶	196 338	13.62¶
CCG 3	216 773¶	26¶	5.28¶	238 193	27.05§
CCG 4**	545 959§	54§	13.24¶	570 234	10.63¶
CCG 5**	140 473¶	18¶	3.49¶	193 410	23.09§
CCG 6	777 024§	103§	18.73§	896 682	16.38¶
CCG 7**	219 981¶	24¶	5.21¶	228 440	9.86¶
CCG 8**	209 101¶	30¶	5.06¶	210 343	15.87¶
CCG 9	218 525¶	22¶	5.22¶	206 440	10.75¶

*England range=2251–1 493 512.

†England average.

‡Index of Multiple Deprivation Score, 2015.

§Above average.

¶Below average.

**Vanguard site.

††England range=5.45–47.39.

CCG, clinical commissioning group.

**Table 2:** Phases of the study and data collected from nine CCGs in the south of England

Phase		Objectives	Main tasks	Data collected
1	Documents: to collect the 2-year Operational and 5-year Strategic plans of CCGs	To determine the accessibility, scale and value of SMS services in the priorities of commissioners	Explore the nature of public accessibility of the forward plans of CCGs, collecting the 2-year and 5-year plans via CCG websites Identify which CCGs have plans available and examine whether and to what extent they mention SM	Publically available 2-year and 5-year plans (via the internet). Content analysis categorised CCGs as high, medium or low profile according to what extent their plans mention SM
2	Interviews: to explore commissioners' conceptualisation of SMS	To acquire an understanding of commissioners' opinions regarding SMS services	Recruit commissioners (through purposive sampling via face-to-face and email contact) to interview	Ten semistructured interviews with commissioners (including Managers, Programme Directors, and GP and lay board members) from six CCGs and one local Strategic Clinical Network. Framework analysis identified the key elements and themes from participants' accounts
3	Observations: to determine what level of input and influence a lay perspective has on commissioning services	To work with patient and public representatives as Service User Researchers to determine what the forward plans and intentions of commissioners mean in practice. How do commissioners make sense of the plans and how is this translated to the public?	Employ SURs (×5) following a formal application process via advertisements sent to voluntary, NIHR and University student organisations. SURs were to be selected with consideration of variety, in terms of: age, gender, health condition, carer status and experience (or lack of) of formal meetings. Facilitate SURs in developing research skills, involvement in project development, taking fieldnotes, debrief sessions, reflective diaries and gathering observations of CCG Governing Body meetings	Field notes and reflective diaries were collated by all researchers during and after the Governing Body meetings as well as debrief notes on their experiences. Governing Body meeting minutes were collected and collated, identifying items relevant to PPI and SMS. These were consolidated with reflective diaries, debrief notes, field notes, the forward plans and interviews, in a workshop with SURs

CCG, clinical commissioning group; SM, self-management; SMS, self-management support; SURs, service user researchers.

**Table 3:** The process of exploring the transparency of NHS purse strings

Phase:	Phase I	Phase II	Phase III
Objective	What is the NHS blueprint?	What is the plan of action and understanding of the blueprint? How do commissioners' conceptualise SM support?	How do commissioners make sense of SMS in practice? What evidence is there of CCGs engaging with the public voice in Governing Body public-facing meetings?
Method	Strategic and Operational plans	Interviews with Commissioners	Observations and fieldnotes

CCG, clinical commissioning group; NHS, national health service; SM, self-management; SMS, self-management support.

Interview participants were sent an information and topic guide before interviews (online supplementary document 1), and written consent was obtained prior to the face-to-face interview. Interviews covered the following topics; commissioners' understanding of SMS, including how they prioritise SMS, whether there are any local drivers for this, how they make decisions about SMS and how their CCG currently supports SMS, as well as whether there are any SMS initiatives currently in development. Commissioners were also asked what changes they have seen in SMS, how they evaluate SMS services, how this feeds back into the commissioning process and what their preferred/desired outcomes for SMS services are. Interviews were audio-recorded, transcribed and anonymised. CCG Governing Body meeting observations involved collecting: field notes, reflective diaries and debrief notes, which were taken by the researchers present at these meetings.

10.1136/bmjopen-2015-010853.supp1 Supplementary data

### Analysis

Operational and Strategic plans were collated and categorised according to the level and content of references to SM using content analysis.^33^ Interview transcripts and fieldnotes were read repeatedly for familiarisation and interview data were coded, using NVivo V.10, with a framework based on our research questions and from reading of relevant policy documents, to describe the data in a literal sense.^34^ Inductive coding allowed us to capture unexpected themes. We examined emerging themes within each interview and compared commissioning practices across the nine CCG localities to identify variation and how SMS services are prioritised. Emerging analytical ideas were explored, discussed and refined in a cyclical process of data collection and analysis.^35^ ^36^ Finally, the presence of SM in the forward plans of CCGs was synthesised with the interview data alongside the published board meeting minutes, reflective diaries and meeting debrief sessions in collaboration with SURs in a workshop.

## Results

Eight CCGs provided access to Strategic plans and seven CCGs provided access to Operational plans (table 4). The CCGs around the south coast were similar to CCGs nationally in terms of accessibility of future plans,^31^ although unlike some CCGs nationally, all of these CCGs provided access to at least one of their forward plans (Strategic or Operational). However, whereas CCGs 1 and 4 produced a combined Strategic and Operational plan together, CCGs 2 and 8 produced a joint Strategic plan, but produced no Operational plan and CCG 9 had no Strategic plan but did have an Operational plan. We conducted 10 interviews, lasting between 30 and 40 min, with commissioners from six of the nine CCGs plus one from the Wessex Strategic Clinical Network. Commissioners from all nine CCGs were invited to participate via email, reminder emails (×2) and telephone contact, but CCGs 7, 8 and 9 provided no response and no indication as to why they would not take part, despite reminder emails. Ten CCG Governing Body meetings were observed (a total of ∼ 30 hours) from five CCGs. Observations at Governing Body meetings were limited to the capacity of the SURs and for CCGs 6, 7, 8 and 9, there was no local SUR availability. Experiences of SURs at CCG Governing Body meetings were collated into Good and Bad practice recommendations (see online supplementary document 2). Of the nine CCG sites, five (CCGs 1, 2, 3, 4 and 5) had data taken from forward plans, interviews and observations, whereas the remaining had data taken from forward plans (except for CCG 6 which also had interview data). Data collection was limited where the CCGs did not respond to invitations to participate in interviews, and the limited capacity of SURs to observe Governing Body meetings.

**Table 4:** Data collected from CCGs in the south of England

	Phase I		Phase II	Phase III
CCG	Strategic plan available?	Operational plan available?	Interview? (N)	Board meeting? (N)
CCG 1	Joint Strategic and Operational plan	Joint Strategic and Operational plan	Yes (1)	Yes (3)
CCG 2*	Yes	No	Yes (1)	Yes (2)
CCG 3	Yes	Yes	Yes (1)	Yes (1)
CCG 4	Joint Strategic and Operational plan	Joint Strategic and Operational plan	Yes (2)	Yes (2)
CCG 5	Yes	Yes	Yes (2)	Yes (2)
CCG 6	Yes	Yes	Yes (2)	No
CCG 7	Yes	Yes	No†	No
CCG 8*	Yes	No	No†	No
CCG 9‡	No	Yes	No†	No
Strategic clinical network	NA	NA	Yes (1)	NA
Total	8	7	10	10

*CCGs 2 and 8 had a joint Strategic plan but no Operational plan.

†Commissioners from all nine CCGs were invited to participate via email, reminder emails (×2) and telephone contact.

‡CCG 9 had no Strategic plan but did have an Operational plan.

CCG, clinical commissioning group. NA, not applicable.

10.1136/bmjopen-2015-010853.supp2 Supplementary data

### Observations of Governing Body meetings

Observations of publically held Governing Body meetings by SURs uncovered how meetings were presented to the public and exemplify the lack of capacity to engage patient and public voices and agendas (online supplementary document 3). These meetings were identified as public-facing meetings and signage at these meetings (online supplementary document 4) represented this as such. Such signs also stated that CCGs are: ‘putting patients at the centre of everything we do’, ‘involve you in the planning and development of services; consult with you on our plans; involve you in decisions about your care; promote choice’, as well as ‘listening to your views and concerns’. Yet, there were no mention of SMS in the Governing Body meetings, no apparent way for patients and the public to engage with decision-making concerning SMS, and no signposting to other decision-making meetings. SURs also noted that lay members on the CCG Board did not seem to be very ‘lay’ in any respect and usually were represented by just one ‘lay’ person. These stark ‘non-findings’ meant that we were unable to do any form of analysis on SMS from the fieldnotes and diaries. At the workshop with SURs, following the Governing Body meeting observations, we reviewed findings from phases I and II. Combined findings allude to a disjunction between aspirations of commissioners and their operationalisation of SMS services. The analysis of the interviews thus focuses on why it is proving hard for commissioners to engage their local population in driving forward and embedding SMS.

10.1136/bmjopen-2015-010853.supp3 Supplementary data

10.1136/bmjopen-2015-010853.supp4 Supplementary data

### To what extent do the CCG plans mention SMS?

**Figure 2:**
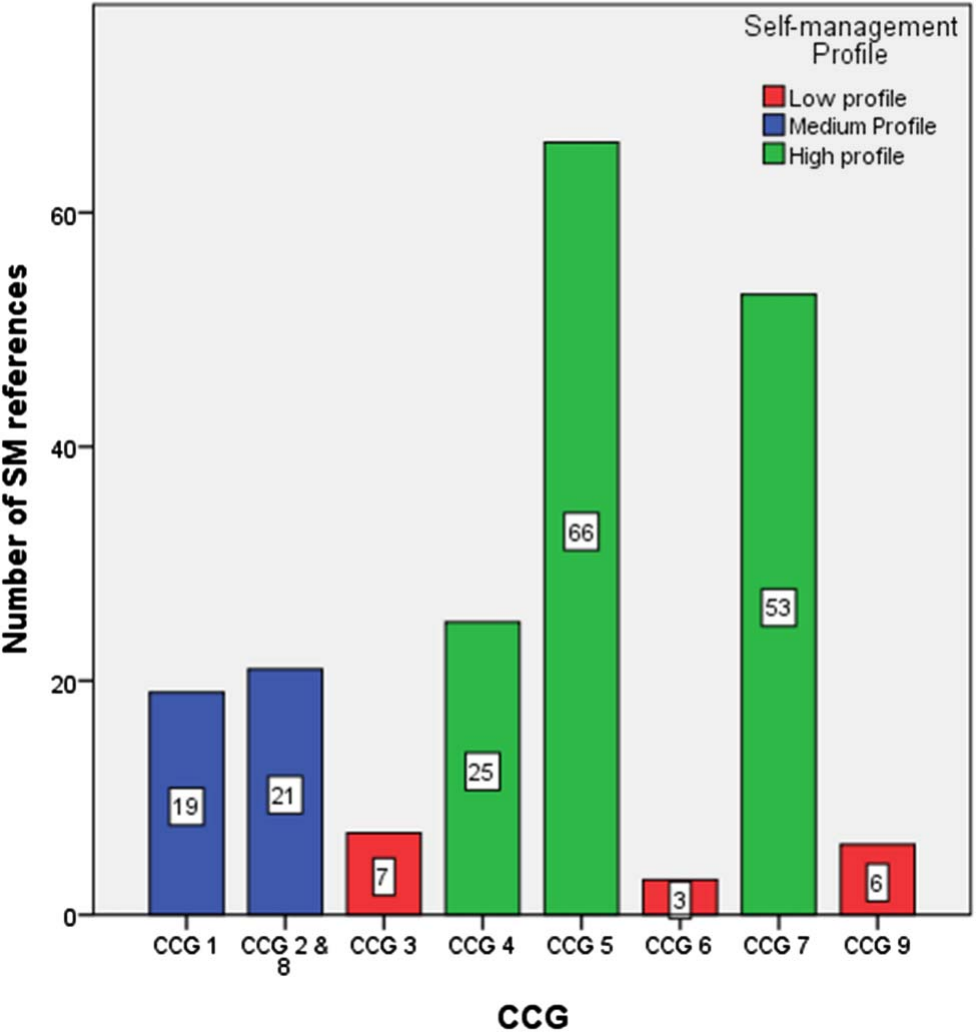
The number of references made to SMS terms in the, available, South of England CCG forward plans (Strategic, Operational or both). CCG, clinical commissioning group; SM, self-management.

A content analysis was undertaken to consider whether the Strategic and Operational plans of CCGs mention SMS (and related terms^i^). SMS (and related terms) was mentioned on 200 different occasions and to varying degrees across the nine CCG's forward plans, ranging from 3 references to 66, with a mean of 25. CCGs were categorised according to whether their plans were regarded as high, medium or low profile (figure 2). The sites which have no affiliation to Vanguard site status are noted for having the lowest number of references to SM terms.

### Commissioners’ conceptualisation of SMS

Three themes were identified from the semistructured interviews with commissioners which are explored below: (1) SMS conceptualisation: a nationally driven agenda; (2) the problem of bringing in new knowledge about SMS into the commissioning process and (3) a lack of capacity to engage patient and public voices and agendas.^ii^

### SMS conceptualisation: a nationally driven agenda

Targeting quality care with a focus on austerity (cost-containment) to maximise value from the NHS budget dominated commissioners' conceptualisation of SMS. Most commissioners used similar ‘key’ terms with reference to SMS, which were commensurate with a ‘top-down’ influence, and expressed as being hard to ‘get right’, suggesting that their understanding of SMS was not inherent, but came from a directive, rather than an individualised personal understanding. Commissioners' expressed understanding was framed by new measures advocated by NHS England such as the Patient Activation Measure (PAM^iii^),^37^ which implies a formulaic simplified means of the evaluation of needs, motivations and abilities of people with LTCs, which they felt would fulfil the remit of a focus on SM;

If you were at Level 1 [of PAM], which would be the lowest, you'd probably maybe be in denial, not think it's your responsibility to manage your health at all and that you would probably expect your GP or secondary care or whoever to actually be dealing with all that stuff for you; it's not your responsibility at all. (Commissioner 5)

Some commissioners' explanations concerning SMS did not indicate that conceptualisation of SMS was acquired from knowledge of local needs of the CCG population. In providing explanations as to how SMS is introduced into the commissioning process, rather than locally driven initiatives, commissioners cite national incentives and refer to guidelines from NHS England (such as the Integrated Care agenda) and the National Institute for Health and Care Excellence (NICE), a public body of the Department of Health which provides national guidance and advice on good practice in healthcare. Although SMS is mentioned as a ‘priority’ for local commissioners, details of local initiatives were notable for their absence. On the face of things, priorities seem to vary between CCG localities but centralised influences, especially those amenable to performance management, are seemingly prioritised. Successes in the development of SMS services tended to be linked to financial incentives such as the Quality and Outcomes Framework (based on pay for performance and a known key motivator in health service provision).^38^ In contrast, if outcome measures and payments for services were to refer to targets unrelated to SMS, this made it difficult, if not impossible, for healthcare professionals to implement and support;

Interviewer: When you're having clinical consultations with patients, to what extent is SM in your mind as you're working through the needs of that person that's sat in front of you?

Respondent: I think you try to keep it there but you often feel that you've got tasks to get through. (Commissioner 3)

There was also a preference for using centrally prescribed evidence with the ‘evidence’ used by commissioners to validate their decisions seemingly derived centrally from NICE and NHS England. Logical pathways were prioritised by commissioners, and measures and outcomes which they felt were ‘tangible’, traditional and safe. Where evaluation of services were referred to, it was reported to be via formal biomedical measures, such as Commissioning for Quality and Innovation, admission rates, amputations and more recently, by the patient activation measure:

As an organisation, one of the key imperatives is to live within your financial means and…we've got our colleagues in Finance who are under tremendous pressure…there's a limited amount of money to be spent and obviously the opportunities for investing in something that might deliver in 10, 15 years is not as attractive to them, as can you do something that sort of changes the balance sheet by the end of this financial year!…I think inevitably, for the hard outcomes, you get drawn back to the national ones there…we may have an opinion on the appropriateness of those there but they are the ones that are measured and so we can't really, you know, move away from those. (Commissioner 7)

### The problem of bringing in new knowledge about SMS into the commissioning process

Most forward plans of CCGs rated as giving SMS ‘high-profile’ (figure 2) declared what SMS services they intended to commission, for example; establishing integrated care teams across the CCG who will work closely with acute trusts to ensure care is delivered promptly in the community and support SMS through appropriate signposting and voluntary sector support, or interventions which use smartphone technology to revolutionise how people can interact with the healthcare system. One CCG was not just aspirational and had already commissioned SMS services, including the employment of a ‘Support Group Development Officer’ utilising a system-wide approach to service design. However, there appeared to be no clear pathway for how such initiatives are brought to the commissioners' table, how they are theorised to be effective ways to enable SM or how they actually come into fruition, other than the link to Vanguard site status (table 1). Such sites have higher incentives for promoting SMS and have more resources to attain this goal.

Additionally, most commissioners acknowledged changes in orientation with a shift towards more patient involvement and empowerment, joint-decision-making and the ‘expert patient’, and moving away from traditional methods of healthcare;

I think there's a huge amount of change in terms of culture, so when I first started in the NHS it was very much basically you manage the patient's condition for them. I think that's completely changed, where a lot of the national guidance has said, ‘Actually you've got expert patients, they know their condition, then actually support that patient, empower that patient to actually manage their condition themselves’ and that just pays dividends…we have to go that way, because…there's not enough resource in terms of doing a hands-on approach for everyone. (Commissioner 15)

However, embedding new knowledge in the commissioning process was more problematic:

If I'm honest, I think it's one of those things we want to do and I'm worried that it will continue to be overshadowed and squeezed out by the demands to meet the insatiable desire for fix-it medicine. (Commissioner 3)

Similarly being able to embed patient-focused agendas and engagement in SMS could be problematic to incorporate into commissioning.

### A lack of capacity to engage patient and public voices and agendas

Preliminary work preceding the in-depth interviews found that 90% of CCGs in England needed to be contacted to gain access to their forward plans (ie, they were not easily, clearly or directly accessible via the website, or were incomplete early drafts or spreadsheets),^31^ suggesting that public accountability and accessibility of plans were not being extensively enacted. This theme of accessibility was replicated in interviews with commissioners;

Interviewer: Are there any local drivers for SMS? Do you get approached by anyone in the community about self-management such as groups, the local cancer groups?

Respondent: Do we get approached? I don't think we get approached; we might approach them…So I think it's about us going to maybe a local charity or a local patient group or you know a local service provider. (Commissioner 13)

The capacity to engage varied between localities. Some areas acknowledged a significant shift towards working with the community and voluntary sector as being part of culture change and priorities moving towards SMS and engaging in their communities, and have developed programmes to help implement this change. But, there was a lack of clarity over how local drivers actually influence the commissioning of services. There is a mention of PPI, but a lack of detail as to how many members of the public and patients are involved, how they are represented in decision-making meetings and, overall, how they input into the decision-making process. While commissioners referred to the latter as drivers, evidence of actual involvement is not as apparent. It seems that rather than communities approaching the CCG with ideas, commissioners' approach selected groups in the communities at their discretion and avoid communicating with people more directly.

## Discussion

The results of this study contribute to the current understandings of how commissioners see, represent and incorporate SMS into commissioning. For SMS to be an integral part of the ‘fully engaged’ scenario, and bring about the greatest gains in public health, services are required that can be adopted by patients. The documentary analysis allowed us to examine how national guidelines on SMS have been interpreted, and then by interviewing commissioners we were able to explore this further. Interviews illuminated how commissioners conceptualise these guidelines, which was found to be fashioned by official terminology and reinforced by group thinking and top-down national agendas. We went on to explore how commissioners' interpretations are then put into practice, and what happens when members of the public approach the only public-facing meeting available to them (CCG Governing Body meetings). In observing such meetings with SURs, it was clear that PPI in SMS decision-making was entirely absent at public-facing meetings. It was found that there were no discussions around a means to ensure SMS services are more personalised and person-centred. Overall, we found that while some CCGs do reference SMS in their plans, and mention that it is an important part of the culture change of the NHS, in practice it is difficult for them to buy into and operationalise SMS if this does not come from a top-down initiative (Vanguard, PAM, etc). Thus, contrary to guidance and policy, CCGs are not implementing services that have come from the needs of the local population. By not offering obvious avenues for patients and the public to engage when they do approach public-facing meetings, it is not clear where a naive member of the public is to go to have their voice heard. In essence, the rates of LTCs and multi-comorbidities are increasing, and as a result, the need for SMS services are too, yet the public voice appears to be lacking in the commissioning of SMS. Where commissioners do want to focus on SMS, they simply do not have the capacity to create these opportunities in their day-to-day work if it does not tie into their traditional, nationally driven, financial incentives.

Effective SMS of LTCs is a key aspiration for improving health outcomes and appropriate utilisation of services for those living with LTCs. Marent *et al*^15^ suggest that including lay perspectives in decision-making could be one strategy to reorient health services towards changing demands in health service provision and patient expectations. However, our initial phases of exploration^31^ found that CCG plans were often inaccessible and that there are regional variations, with less wealthy areas at risk of not being involved with the commissioning of their health services. In our current study, we have found that there is also variation about how much SMS is mentioned or prioritised in the forward plans, by individual commissioners and in Governing Body meetings. Some areas are clearly prioritising SMS in their key outcomes more than others and implementing a variety of SMS resources. Such sites are more often than not ‘Vanguard sites’; those awarded with higher incentives and means to attain this goal. With financial drivers and structural limitations being noted by commissioners as the key drivers as to what actually gets commissioned in practice, and alluding to a commissioning process which is often fragmented, this increased financial incentive through Vanguard status appears to give an artificial advantage to the selected sites in implementing SMS.

Currently, CCGs are measured on their adherence to national directives and financial incentives, yet it is evident that effective SMS demands more than an order from NHS England. Commissioning decisions are made with reference to ticking the boxes of key biomedical outcome measures, which are often incongruent to measures which reflect improved SMS for patients (ie, self-efficacy, shared decision-making, health-related quality of life and psychological well-being). Improved SM should improve biomedical outcomes and not the other way around. In essence, CCGs are performance-managed against centralised drivers, especially in terms of austerity. Procedural and biomedical markers (eg, the percentage of patients who turn up to outpatient appointments), which can be directly linked to financial impacts on the service, are what gets measured, with a strong sense of lip service to national priorities which are hard to get into practice on the front line. If outcome measures and payments for services refer to targets unrelated to SMS, this makes it difficult, if not impossible, for healthcare professionals to implement support. Where SMS services are being actively commissioned these have been introduced through top-down (rather than locally driven) initiatives, that is, the Integrated Care agenda, national ‘Vanguard’ sites and, more recently, NHS England's promotion of the PAM.^39^ Using PAM requires purchase of a licence by CCGs in order to use it to assess patients' engagement with their health. It is a way to measure the population's level of ‘activation’ regarding SM rather than an intervention to support SM, and it is also not a tool which has been designed or developed through engagement with patients or the public. It is through a focus on formulaic evidence and minimal and poor PPI engagement that the formulation of SMS services have not, to date, progressed further.

### Relevance of study with regard to wider literature/comparison with previous studies

Despite the rhetoric of ensuring services are designed around patients' needs, we found that patients and the public were not engaged in commissioning in meaningful ways and their voice was, almost entirely, absent. CCG Governing Body meetings are held in front of a public audience, but are not ‘public meetings’ in the sense of participation. Whilst CCG's propose to be more accountable to the public, Governing Body meetings remain the principle forum for direct public engagement, but provide few opportunities for CCGs to learn from the experiences of patients and the public. This resonates with Smith *et al*'s^40^ study on commissioning high-quality care for people with LTCs, in primary care trusts, shortly before the restructuring of commissioning which found that commissioning meetings and workshops tended to be more of a ‘ritual’ rather than fulfilling the purpose and potential of such gatherings to involve people with specific interests to deliver outcomes. The results presented here also resonate with Checkland *et al*'s^22^ exploration of accountability in the new CCGs, in so far as questions could be asked by the public at the beginning of board meetings, but not in response to matters raised during the meeting. While the CCG sites explored in Checkland's study expressed intentions to set up additional forums for patients and the public, such ‘additional forums’ were not made accessible to the SURs in the current study. So where else do CCGs expect to be held accountable? CCG board meetings are CCG's public-facing meetings, and their opportunity to interact with their public, and to be accountable, but with no known access to SMS decision-making meetings and subgroups, the standing of real transparency and accessibility to SMS decision-making in CCGs is questionable.

No research, to date, has investigated the perspectives of commissioners on their desired outcomes of SMS services,^41^ despite their key role in commissioning patient-focused SMS services. Efforts that feel more like a tick box exercise for accountability, rather than a genuine pursuit of the public and patient perspective, can be entirely fruitless, seeding a feeling of suspicion and distrust.^23^ Contrary to the prevailing one-size-fits-all model of lay involvement, which does not tailor to the needs of particular demographics, Armstrong *et al*^42^ have identified specific strategies to help ensure that patient involvement can realise its full potential. They recommend a participative approach, laid out beforehand in strategic planning with a clear agenda, although most CCGs do not currently have this capacity. This study adds to the literature around the importance of SMS and that for effective LTC management, good SMS that people can build into everyday life is key, while providing evidence for the rarely sought, understood or known commissioner view on what SM is or how they actually involve the patient and public voice to inform their decisions. It highlights that without in-depth knowledge on the existing preferred outcomes of all stakeholders, there is a risk that support services for SM will be commissioned that have, potentially, limited impact on their target population.^41^

### Implications

CCGs charged with commissioning services for LTCs reflect the health policy priority of including and providing improved provision for SMS services. This study allows us to understand the gaps present in the commissioning of SMS services, and where CCGs can target to begin to achieve their ambitious 5-year plans. This can be described in terms of the ‘third translational gap’,^43^ considering the integration of healthcare as it occurs at the level of the individual patient within the wider context of their lives. A focus of work looking at implementation in community and domestic settings brings to the fore a commitment to working with patients and the public.^43^ Understanding what the commissioning landscape currently holds for SMS offers an opportunity to target areas for improvement and implement meaningful strategies and innovations for improvement.^10^ These areas include improvements to the health service overall by improving patients' health and well-being^8^ ^16^ and at a system level.^8–11^ As an outcome of all of these, there are reduced health systems costs.^8–11^

There are instances where commissioners are trying to fulfil this drive for openness, accessibility, transparency and patient feedback, but where SMS starts as a priority in CCG plans, it becomes less obvious in the day-to-day work of commissioners. Attending Governing Body meetings from the perspective of the people, the CCG is striving to serve left fundamental questions regarding how the CCG is actually listening to the patient voice. The ‘pressing’ focus, in reality, is on financially driven imperatives, meaning that putting SMS into practice becomes the hurdle at which most commissioners' fall.

This study highlights where CCG aspirations and operationalisation do not align, and draws attention to where intentions are not being put into practice—effective SMS which is developed from the bottom-up. While the culture of the NHS is moving away from a medical model to a more person-centred model, the desire for SMS cannot be met without a structure which allows the flexibility for adaption to local needs, so that changes can be incorporated to enable increased capacity to facilitate corporation. The imperative of patients' voice and choice has taken on reinforced authority in the light of failures in fundamental care^44^ ^45^ and is thus worthy of exploration in newly established organisations responsible for the commissioning of services. In relation to SMS, where patients and the public are coproducers and providers of the capacity to enact support, lay involvement in policymaking and commissioning has increased in salience. If CCGs are willing to collaborate and learn from the experiences of their patients, then they can set in motion the implementation of services which are able to effectively address the needs of the people using their services, turning guidance and policy into actual experience.

## Footnotes

i Related terms; ‘self-management’, ‘Manage their own condition’, ‘Self-help’, ‘Self-care’, ‘Coproduction’, ‘House of Care’, ‘Expert patient’, ‘Self-medicate’, ‘Self-monitor’ and ‘Patient activation’.

ii Quotes from interviews are provided below where these are typical of responses across the sites, or where they illustrate a particular issue well.

iii The PAM is a tool for measuring the level of patient engagement in their healthcare.

iv The south of England is more affluent than other UK regions but includes areas which are more deprived (according to the IMD 2015 where lower than average figures indicate more affluence), and a variation of population sizes, practice sizes and revenue etc.
