# Supplementary material for: Commissioning of self-management support for people with long-term conditions: an exploration of commissioning aspirations and processes
Source: BMJ Open. 2016 Jul 15;6(7):e010853. doi: 10.1136/bmjopen-2015-010853 (PMC4964251; doi:10.1136/bmjopen-2015-010853)
Supplement: Supplementary data [file bmjopen-2015-010853supp1.pdf]

Supplementary information 1

Interview Schedule: Commissioners

Welcome and Introductions

Researcher to welcome participant(s), and to introduce self and the briefly say about the study and the aim of the interview.

- Topic of self-management
- Concerned with hearing *your* views regarding patient's self-management of their health conditions, in particular, what do you see as the important outcomes of this.
- Reminder of research rights- right to withdraw, pause or not answer certain questions.
- Interview will be tape recorded, transcribed etc.

Learning about the participant

- Job title
- Nature of job role
- Length of time in current position and in the field of XX
- How does this relate to either one of the conditions / or self-management
- Do you have a brief to specifically commission services to support self-management?

Understanding Self-Management

- What is your understanding of self-management? Self-management support? How would you describe self-management support?
- How is self-management prioritised in the commissioning process? Are there any local drivers for this?
- How does your CCG currently support self-management? Are there any initiatives currently in development to support SM?
- What changes have you seen in SM support over your time in the NHS?

Outcomes of Self-Management

- How much do you prioritise self-management in the context of all of your other demands?
- How do you make a decision regarding what SM services to commission?
- What are the preferred/desired outcomes of any services for SM?
- How are services for SM support evaluated?
- What are the things you are looking for when evaluating SM related services?

- How does the evaluation feedback into the commissioning process?

End

- Anything else to add?
- Any questions for us?
