# Supplementary material for: Commissioning of self-management support for people with long-term conditions: an exploration of commissioning aspirations and processes
Source: BMJ Open. 2016 Jul 15;6(7):e010853. doi: 10.1136/bmjopen-2015-010853 (PMC4964251; doi:10.1136/bmjopen-2015-010853)
Supplement: Supplementary data [file bmjopen-2015-010853supp2.pdf]

Supplementary document 2: Good & Bad Practice recommendations from service-user researcher's observations at CCG board meetings

**Good Practice**	
**Theme**	**Feedback**
Engagement	Interactive and engaging meetings which provided Public seminars, and encouraged discussion, as well as succinct board papers.
Being welcomed	Engaging, passionate and friendly Chair (i.e. where they acknowledged the effort of the staff behind the reports), gives good impression
	Demonstrable enthusiasm/dedication from board members
	Use of humour – made the CCGs appear "more human"
Agenda items	Time for questions to the Board – some opportunity for engagement
	Clear, succinct agendas and papers, including glossary or acronyms, colour coded papers/packs = helpful, use of tabs to separate agenda items
	Chair giving very brief summary of next agenda in 'lay terms'
Environment	Clear sound system, in accessible venue and location
	Clear signage, information and advertisement of meeting
	Plentiful refreshments
What is missing (questions that arose)	What is the purpose? Accountability?
	Big issue around non evidence of self-management support
	Where do you go, as a member of the public who wants to have a say/influence?
**Bad Practice**	
Intimidating	Needs less intimidating atmosphere and less daunting language, and smaller, colour coded papers
	Can feel awkward (it was like they weren't expecting us"), feeling like

Boring (due to being; dull, difficult to understand, difficult to follow, "medic" speak)	you stand out, "Why are you here" (common question posed by board members to SURs) could be intimidating, Off-putting signs i.e. private meeting<br><br>First meeting difficult to follow and understand (Can feel like a secret code/language that you have to learn), agenda too long and difficult to follow, Board members speak in medic jargon, or in a monotone<br><br>Deluge of financial info (too much to assimilate)<br><br>When are decisions actually made? Most of the meetings appeared to be conducted beforehand – rubber stamping exercise
Practicalities	No refreshments<br><br>No signage – where to go? No parking (troublesome and costly to attend)
