# Supplementary material for: Commissioning of self-management support for people with long-term conditions: an exploration of commissioning aspirations and processes
Source: BMJ Open. 2016 Jul 15;6(7):e010853. doi: 10.1136/bmjopen-2015-010853 (PMC4964251; doi:10.1136/bmjopen-2015-010853)
Supplement: Supplementary data [file bmjopen-2015-010853supp3.pdf]

Supplementary document 3. A summary of observations from CCG Commissioning board meetings Five SURs and three university researchers observed ten CCG board meetings. The following is a summary of their field notes, reflective diaries and discussions;

Point 1: Little evidence of SMS or public engagement

In practice there was little evidence of PPI engagement demonstrable to the public. Items related to SMS or PPI were often not present in CCG board meeting agendas which covered typical standing business items (e.g. various performance, finance and governance reports). Only where the Chair of the Board made specific attempts to include and encourage public engagement, or where public seminars were held relating to specific matters of interest to the public, was this not this case. In these instances accountability was enhanced.

Point 2: No access to decision making; Board meetings are for "rubber stamping"

There was a lack of opportunity to exercise meaningful contribution to the decision making processes of the commissioning of health services, either during public board meetings or through being signposted to other forums of involvement outside of these meetings, and PPI representation was minimal to nil. Personal narratives from commissioners along with internalised and collectively reinforced tacit guidelines dominated decisions, rather than referring to insight gained from patients. Public board meetings appeared to be more an exercise of 'rubber-stamping' decisions made at other, non-accessible meetings rather than an actual forum for decision making;

"Nothing was spoken about the decision making process – there was just consensus during the meeting, it was not about the process." (Service User Researcher 5).

Point 3: Jargon filled, finance focused agendas

SURs repeatedly reported finance-laden agendas and extensive discussions over financial matters, governance and performance reports/risk;

"decisions are not being made in regards to SMS services, SMS was not brought up at all. I felt that finance dominates most decisions, or at least did on this occasion. In the meeting papers there were concerning indicators for the diabetes services, but these were not mentioned at all!" (Service User Researcher 1)

Point 4: Lengthy, unfriendly meetings

Board meetings consisted of a tightly condensed agenda, which rarely kept to time, varying from 2 to 5 hours in length, and sometimes difficult to locate. There was much reflection from SURs over

the purpose to these meetings and collective remarks on the goldfish bowl effect that the CCG board meeting appears to be.

"Additionally, there appeared to be little consideration for where members of the public would sit… It was almost as if they hadn't expected us."(Researcher 3)

Point 5: Meaningful user-involvement?

Commissioning of SMS was not obvious in the day to day practice of commissioning and local voices are often not engaged, suggesting that local voices were not of precedence in contributing to commissioning decisions;

"My overall impression from the board meeting was that it is for themselves - the board members, not the public. There were very few instances where they brought it back to where the agenda items affect people – and they could've done more about this. The patient perspective was not brought up / thought of" (Service User Researcher 1)
