# Supplementary figures and images for: Commissioning of self-management support for people with long-term conditions: an exploration of commissioning aspirations and processes
Source: BMJ Open. 2016 Jul 15;6(7):e010853. doi: 10.1136/bmjopen-2015-010853 (PMC4964251; doi:10.1136/bmjopen-2015-010853)

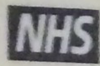

**PUBLIC BOARD  
MEETING HERE  
TODAY**

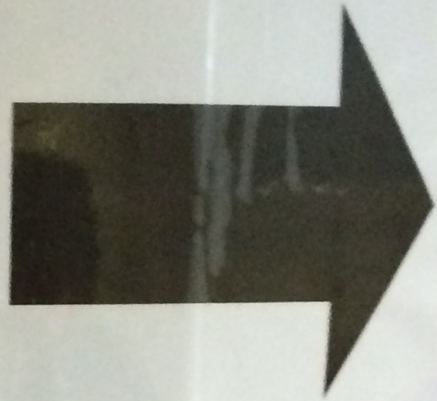

Supplement: Supplementary data [file bmjopen-2015-010853supp4.pdf]
